# Association between common mental disorders and the severity of dysmenorrhea among female medical students at the University of Ibadan, Nigeria

**DOI:** 10.1371/journal.pgph.0004492

**Published:** 2025-04-23

**Authors:** Yusuf Olatunji Bello, Oluwabunmi Victoria Adeyeye, Mary Mofiyinfoluwa Adeyeye, Olajumoke Aishat Oladosu, Oluwagbemisola Motunrayo Oderemi, Nasirat Ibukun Akinlade, Millicent Magdalene Maduka, Gloria Onyinyechi Madu, Victor Makanjuola, Jibril Abdulmalik, Imran Oludare Morhason-Bello

**Affiliations:** 1 Department of Statistics, Faculty of Science, University of Ibadan, Oyo, Nigeria; 2 Institute for Advanced Medical Research and Training, College of Medicine, University of Ibadan, Oyo, Nigeria; 3 Department of Medicine and Surgery, Faculty of Clinical Sciences, College of Medicine, University of Ibadan, Oyo, Nigeria; 4 Department of Medicine and Surgery, Faculty of Clinical Sciences, College of Medicine, Abia State University, Abia, Nigeria; 5 Department of Psychiatry, Faculty of Clinical Sciences, College of Medicine, University of Ibadan, Oyo, Nigeria; 6 Department of Obstetrics and Gynaecology, Faculty of Clinical Sciences, College of Medicine, University of Ibadan, Oyo, Nigeria; St John's Medical College, INDIA

## Abstract

This study examines the association between common mental disorders, such as anxiety, depression, stress, and low self-esteem, and the severity of dysmenorrhea among female medical students at the University of Ibadan, Nigeria. This cross-sectional study was conducted among 171 female medical students that were menstruating regularly. Data were collected with a structured questionnaire validated by expert independent observer. Common mental disorders scores were computed using standardized assessment tools including GAD- 7 Anxiety Scale, Beck Depression Inventory, Perceived Stress Scale, and Rosenberg Self-Esteem Scale. The correlation coefficient was used to assess the relationship between each common mental disorder and the severity of dysmenorrhea. The Quantile-based G-computation (QGC) and Bayesian Kernel Machine Regression (BKMR) were used to evaluate both the relative and collective influence of the four common mental disorder scores on the severity of dysmenorrhea. There was a positive association between common mental disorders and severity of dysmenorrhea except self-esteem that was negatively associated. An increase of 1.17 (95% CI: 0.28 – 2.06) in the severity of dysmenorrhea per quartile difference was observed for the combined influence of the four common mental disorders. Anxiety had the highest relative influence on the severity of dysmenorrhea (weight: 0.36), followed by depression (weight: 0.31) and self-esteem (weight: 0.24). The collective influence of the four common mental disorders on the severity of dysmenorrhea was greater among participants with unsatisfactory relationships with their parents (*β* = 1.25) compared to those with satisfied relationships (*β* = 1.01). The study demonstrated a collective increase in the influence of the four common mental disorders on the severity of dysmenorrhea among female medical students.

## 1 Introduction

Primary dysmenorrhea refers to cramps arising from the uterus during menstruation without any associated pelvic pathology. This condition typically manifests in females aged 25 years or younger. Studies have indicated that between 25% to 95% of female students experience dysmenorrhea [1–5]. According to a recent study we conducted on the prevalence and risk factors for dysmenorrhea among medical students at the University of Ibadan in Nigeria, 96.45% of female students reported having this kind of pain every month [6]. Previous research has also demonstrated that students who experience dysmenorrhea frequently miss school, which lowers their quality of life. Students with dysmenorrhea were found to miss school between 14% to 51% of the time [7]. Furthermore, it has been found that during dysmenorrhea periods, the rate of class attendance decreases by 29% to 50% [2].

Several factors have been well-studied in relation to dysmenorrhea, including low socioeconomic status [8,9], age [10,11], religion [12], satisfied relationship with parents [6], smoking [13], body mass index [11], alcohol consumption [14] and family history of dysmenorrhea [15]. Furthermore, emerging evidence suggests that psychological factors, such as common mental disorders, could also influence the severity of dysmenorrhea [16]. This connection may be due to shared physiological mechanisms, such as hormonal fluctuations affecting both menstrual cycles and mood, as well as stress-related changes in pain perception and inflammatory biomarkers [17].

Dysmenorrhea and common mental disorders exhibit a bidirectional relationship [16]. In simpler terms, monthly menstrual pain may worsen if a woman is depressed, anxious, or stressed, and vice versa. Research has indicated that depression amplifies the impact of pain on social and occupational functioning while also reducing the likelihood of a positive response to medical intervention. Moreover, it was discovered that people who experienced migraines or other types of headaches had higher anxiety levels [18]. It is believed that anxiety and depression, which have been linked to various pain conditions are also associated with dysmenorrhea [19]. Menstrual cycle disorders and dysmenorrhea are aggravated by emotional and behavioral disorders. According to a study on young women, anxiety and depression can both affect the severity of dysmenorrhea [20]. These interconnections are particularly relevant among university-aged women, as this group is susceptible to both academic pressures and mental health challenges, potentially intensifying the effects of dysmenorrhea [21,22]. This study assessed the association between common mental disorders, such as anxiety, depression, stress, and low self-esteem, and the severity of dysmenorrhea among medical students. Investigating this association in medical students at the University of Ibadan, Nigeria, provides a valuable context for examining these relationships in an academically rigorous environment and will help us develop a better understanding of the challenges faced by medical students, as well as their potential effects on health and academic achievement. This could lead to improved management outcomes for dysmenorrhea through the inclusion of psychological or psychotherapeutic interventions among medical students and women in the general population.

## 2 Materials and methods

### 2.1 Study design

This was a cross-sectional study conducted among female medical students at the College of Medicine, University of Ibadan (UI). The University of Ibadan, established in 1948, is Nigeria’s oldest degree-awarding institution. The College of Medicine within the university has a diverse student’s population from various regions, ethnicities, and backgrounds in Nigeria.

### 2.2 Study setting

Data were collected from female medical students (years 1–6), aged 17 – 30 years and with a history of regular menstruation at the College of Medicine, UI. Female students with abnormal bleeding, pelvic inflammatory diseases, lower abdominal pain, or pelvic pain due to other causes and those who refused to consent were excluded from the study.

### 2.3 Sampling technique

According to the students’ records, there were 40, 68, 93, 63, 58, 57, and 40 female students at each level from Year 1 to Year 6 at the College of Medicine, UI, respectively. This made a total of 419 female students. A group of students had a one-year academic delay due to labor union industrial actions. To ensure representative sampling and minimize potential bias, a stratified random sampling technique was used. Each academic year was considered a stratum, and the number of participants selected from each year was proportional to the total number of female students in that year. Within each stratum, students were randomly selected to participate in the study.

### 2.4 Sample size

The required sample size was calculated to be 167 using the sample size formula for cross-sectional design [23–25]:


$$n=\frac{{\left(Z_{1-\frac{\alpha }{2}}\right)}^{2}p\left(1-p\right)}{d^{2}}$$

Where:

*n* is the required sample size

$Z_{1-\frac{\alpha }{2}}$ is the critical value and a standard corresponding level of confidence at 95%

*p*is the prevalence of dysmenorrhea from a previous study 87.6% [26] and

*d* is the margin of error or precision of the study at 5%.


$$n=\frac{{\left(1.96\right)}^{2}*0.876\left(1-0.876\right)}{{\left(0.05\right)}^{2}}=167$$

Four additional participants were added to the sample size to account for nonresponse bias, making a total of 171 participants included in the study. This conservative approach was taken, considering the participants were learned medical students.

### 2.5 Study instrument and data collection

A structured questionnaire consisting of six sections and closed-ended questions was used to collect data on the severity of dysmenorrhea, mental health, and socio-demographic variables. The development of the questionnaire was informed by a comprehensive review of the literature on common mental disorders and dysmenorrhea, which highlighted key disorders such as anxiety, depression, stress, and self-esteem. This literature review also guided the selection of the validated tools such as GAD-7 Anxiety Scale, Beck Depression Inventory, Perceived Stress Scale, and Rosenberg Self-Esteem Scale that have been previously used and validated among medical students [27–30].

The questionnaire was further refined through consultation with experts in gynecology and psychology at the College of Medicine, University of Ibadan, to ensure content validity and cultural sensitivity for the study population. These experts reviewed the items for clarity, relevance, and appropriateness and deemed the content safe for all categories of female medical students. Any disagreements or differing opinions among experts were discussed and resolved through consensus to align with the study’s primary objectives.

After, another independent specialist (obstetrician and gynecologist) reviewed the questions for the second time before a pilot study was conducted among female students in another faculty at the University of Ibadan. The pilot study involved 30 female students. Key findings included identifying areas in the questionnaire that needed refinement for clarity and conciseness, particularly in the section on menstrual patterns. This feedback helped improve the reliability of the main study and ensured that participants clearly understood the questions.

The data collection process began on 26th August 2022 and concluded on 27th September 2022. Potential participants were selected from a pool of sampling frame of medical students in each academic level. The faculty members of the institution were not involved in the data collection process to avoid putting the students under any undue pressure or influence their decisions. The student research assistants involved in the study were trained on the data collection process, and the questionnaire was anonymized to minimize bias. The student research assistants distributed the questionnaire to selected students until the desired sample size was achieved.

### 2.6 Data management and analysis

The study examined four common mental disorders: anxiety, depression, stress, and self-esteem. Anxiety scores were evaluated using the GAD-7 Anxiety Scale. To calculate these scores, responses such as “not at all,” “several days,” “more than half the days,” and “nearly every day” were assigned values of 0, 1, 2, and 3, respectively. The values for all the seven questions were then added together. Cutoff points for mild, moderate, and severe anxiety were set at 5, 10, and 15, respectively. Participants were categorized as having an anxiety disorder if their score was higher than 15 [31].

The study measured the degree of depression among participants using the Beck Depression Inventory (BDI) scale. This inventory assesses depression through 21 symptoms and attitudes. The items were rated from 0 to 3 to indicate their level, and the results were summed linearly to get a score ranging from 0 to 63. Higher scores indicate more severe symptoms of depression. Participants with a depression score exceeding 20 were considered to be experiencing depression [32].

The Perceived Stress Scale (PSS) was used to evaluate participants’ stress levels by evaluating how stressed they feel about various aspects of their lives. The items were designed to capture the irregular, chaotic, and hectic nature of participants’ lives. The scale includes straightforward questions about current stress levels experienced. A total PSS-10 score ranging from 0 to 40 is possible, with higher scores indicating a greater level of stress. Participants scoring higher than 26 on the PSS were classified as experiencing high perceived stress [33,34].

The self-esteem of participants was assessed with a 10-item Rosenberg Self-Esteem Scale (RSES). The scale assessed overall self-esteem by evaluating the positive and negative sentiments of individuals. The scores range from 0 to 30, where a higher score denoted low self-esteem and lower score indicated high self-esteem. Participants who scored 15 and above on the RSES scale were considered to have low self-esteem [35].

The severity of dysmenorrhea served a dependent outcome. The severity of dysmenorrhea was measured using a numerical rating scale from 0 to 10, where 0 represents no pain and 10 represents the worst pain ever imagined. Other information gathered includes the history of dysmenorrhea, sociodemographic characteristics, menstrual patterns, and family history of dysmenorrhea.

Descriptive analyses were performed to summarize participants’ sociodemographic characteristics and common mental disorders. The computed scores for each of the common mental disorder were centered and standardized to have a mean of 0 and a standard deviation of 1 using Z-score [36]. The Spearman correlation coefficient was employed to assess the relationship between the scores of each common mental disorder and the severity of dysmenorrhea.

The Quantile-based G-computation (QGC) and Bayesian Kernel Machine Regression (BKMR) were employed to assess the relative contributions and collective influence of the four common mental disorder scores on the severity of dysmenorrhea. Identified risk factors associated with dysmenorrhea (students’ monthly allowance, menstrual cycle length, accommodation type, religion, and relationship with parents) were included as covariates. These five variables were selected based on their relevance to the study population and their potential influence on the severity of dysmenorrhea. Furthermore, the QGC and BKMR analyses were stratified by students’ monthly allowance, relationship with parents, and type of accommodation. The level of statistical significance was set at 0.05. Missing observations were included in the descriptive analyses but were excluded from the QGC and BKMR. Descriptive analyses were conducted using STATA (StataCorp L.L.C.), while R version 4.4.0 (R Core Team, 2024 was used for the QGC and BKMR analyses.

### 2.7 Quantile‑based g‑computation (QGC)

In QGC, each common mental disorder’s scores were transformed into quantized form, denoted as $MDisorder_{i}^{q}$, and coded as 1, 2, 3, or 4. These scores were then fitted to a linear model represented as follows:


$$Y_{\text{dys}}=\beta_{0}+\psi \overset{4}{\underset{i=4}{\sum }}w_{i}MDisorder_{i}^{q}+\epsilon_{j}$$

$$Y_{\text{dys}}=\beta_{0}+\overset{4}{\underset{i=4}{\sum }}\beta_{i}MDisorder_{i}^{q}+\epsilon_{j}$$

Where $\beta_{i}$ is the effect size for the ithMDisorder, $\epsilon_{j}$ is the error term and $\psi =\overset{4}{\underset{i=4}{\sum }}\beta_{i}$represents the change in severity of dysmenorrhea per one-quartile change of all four common mental disorder scores after controlling for covariates.

When the influence directions are the same in the selected common mental disorder, the weight for the kth disorder is defined as $w_{k}=\frac{\beta_{k}}{\sum_{i=1}^{4}\beta_{j}}$. The weights are defined for each influence direction, with positive directions totaling 1.0 and negative directions totaling −1.0. The weight represents the percentage of the influence of each specific common mental disorder with the same direction and is utilized in QGC to capture the relative contribution. In collective influence, a larger weight denotes a greater influence. Point estimates and 95% confidence intervals (95% CI) for QGC analyses were obtained using the R package QGCOMP.

### 2.8 Bayesian kernel machine regression (BKMR)

The relative influence of the four common mental disorder scores on the severity of dysmenorrhea was also investigated using Bayesian Kernel Machine Regression (BKMR) with a Gaussian kernel. The BKMR model equation for this study can be expressed as follows:


$$Y_{dys}=h\left(MDisorder_{i}\right)+Covariate_{i}^{T}\beta +\epsilon_{i}$$

Where $MDisorder_{i}=\left(MDisorder_{i1,}MDisorder_{i2},MDisorder_{i3},andMDisorder_{i4}\right)$ is a vector comprising all four common mental disorder scores for the ith participant. $Covariate_{i}^{T}$ is the matrix of the covariates, *β* is the vector of corresponding coefficients for covariates and $\epsilon_{i}$ is the error term. The influence of each common mental disorder on the severity of dysmenorrhea was defined by setting all common mental disorder scores to their median and adjusting for the covariates. In BKMR, a Gaussian kernel was employed within a Bayesian framework to specify the unknown disorder influence h() and compute the posterior inclusion probability (PIP). After controlling for covariates, the estimates for the PIP were defined as the change in the severity of dysmenorrhea when all four common mental disorder scores were fixed at their 25th and 75th percentiles.

For each BKMR model, a total of 50,000 iterations were conducted to fit the Markov Chain Monte Carlo (MCMC) sampler. The relative influence in BKMR was quantified by the PIP, which reflects the extent to which the severity of dysmenorrhea was influenced by each common mental disorder. A higher PIP indicates a greater influence. The PIP for BKMR analyses and visualizations were obtained using the R package BKMR.

### 2.9 Ethical consideration

The study obtained ethical approval from the University of Ibadan/University College Hospital (UI/UCH) Ethics Committee, with reference number UI/EC/22/0266. Each participant read an information leaflet that described the study in detail and signed a written informed consent prior to participation. The study did not include minors, with the minimum age of participants being 17.

## 3 Results

In this analysis, data from 171 medical students were used. The mean age was 22.1 ± 0.2 years. The majority (96.5%) experienced dysmenorrhea, with 36.6% describing it as severe. Among the total participants, 72 (47.1%) did not receive sufficient monthly allowance from their parents, or guardians. Majority of participants were adjudged to be free of anxiety (86.6%), depression (90.6%), high perceived stress (86.6%), and had a high self-esteem (89.2%) (Tables 1 and 2).

**Table 1:** Distribution of participants’ characteristics and common mental disorders.

Variables	Total (N = 171) n (%)	Dysmenorrhea^1^	
**Yes** **n (%)**	**No** **n (%)**
**Age [Mean (Sd)]**	22.1 (0.2)	22.09 (0.2)	21.8 (0.7)
**Study program**			
Medicine and Surgery	143 (83.6)	136 (83.4)	5 (83.3)
Dentistry	28 (16.4)	27 (16.6)	1 (16.7)
**Accommodation** ^**2**^			
In campus	143 (85.1)	135 (84.4)	6 (100)
Alone off campus *(rented apartment)*	19 (11.3)	19 (11.9)	0
Off-campus with family	6 (3.6)	6 (3.8)	0
**Religion**			
Christianity	153 (89.5)	145 (89.0)	6 (100)
Islam	18 (10.5)	18 (11.0)	0
**Ethnicity** ^**1**^			
Yoruba	115 (68.1)	112 (69.1)	2 (33.3)
Hausa	2 (1.2)	2 (1.2)	0
Igbo	36 (21.3)	34 (21.0)	2 (33.3)
Others	16 (9.5)	14 (8.6)	2 (33.3)
**Marital Status**			
Single	167 (97.7)	159 (97.6)	6 (100)
Married	2 (1.2)	2 (1.2)	0
Separated	2 (1.2)	2 (1.2)	0
**Parent Relationship** ^**2**^			
Satisfied	149 (88.7)	143 (88.3)	6 (100)
Indifferent	9 (5.4)	9 (5.6)	0
Unsatisfied	10 (6.0)	10 (6.2)	0
**Monthly Allowance** ^**3**^			
Insufficient	72 (47.1)	71 (48.3)	1 (20.00)
Sufficient	81 (52.9)	76 (51.7)	4 (80.00)
Menstrual cycle length [Mean (Sd)] *(Duration from the first day of the last menses to the time of the next one)*	27.7 (0.4)	27.9 (0.4)	21.4 (4.7)
**Anxiety Disorder** ^**4**^			
No	142 (86.6)	137 (87.3)	5 (83.3)
Yes	22 (13.4)	20 (12.7)	1 (16.7)
**Depression Disorder** ^**5**^			
No	144 (90.6)	139 (90.3)	4 (100)
Yes	15 (9.4)	15 (9.7)	0
**Perceived Stress** ^**4**^			
Low perceived stress	142 (86.6)	137 (87.3)	5 (83.3)
High perceived stress	22 (13.4)	20 (12.7)	1 (16.7)
**Self-esteem** ^**6**^			
High self-esteem	148 (89.2)	140 (88.6)	6 (100)
Low self-esteem	18 (10.8)	18 (11.4)	0

1 – 2 missing, 2 – 3 missing, 3 – 18 missing, 4 – 7 missing, 5 – 12 missing, 6 – 5 missing.

**Table 2:** Descriptive statistics of common mental disorders scores.

common mental disorders	Minimum	Mean	Standard deviation	Maximum
Anxiety	0	4.96	4.77	21
Depression	0	8.26	8.10	44
Stress	4	18.32	7.33	35
Self-esteem	11	20.70	5.28	30
Dysmenorrhea severity	1	5.54	2.14	10

**Fig 1:**
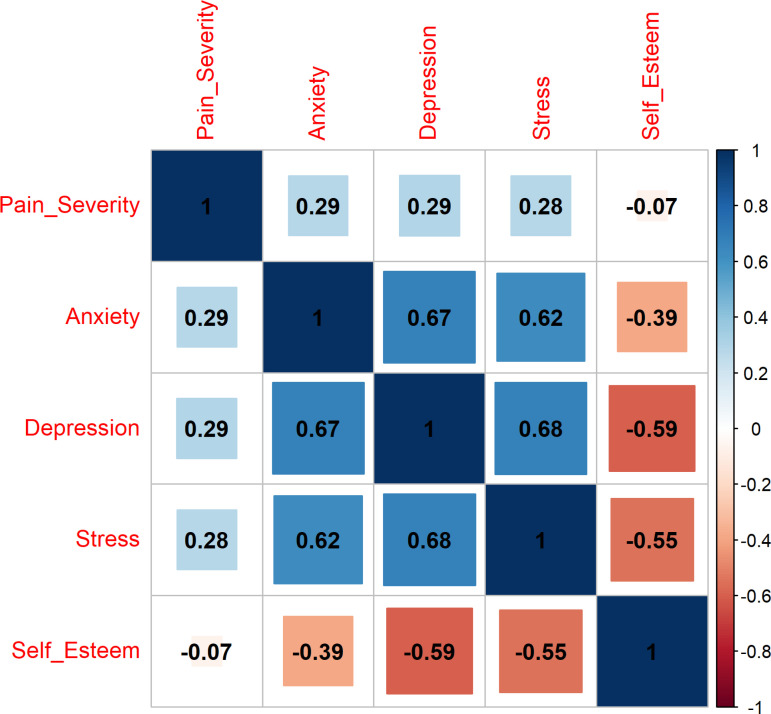
Spearman correlation between the severity of dysmenorrhea and each mental health disorder scores.

The Spearman correlations between the severity of dysmenorrhea and each of the four common mental disorders are shown in Fig 1. Anxiety and depression showed the strongest relationship (R = 0.67). Although the association was weak, anxiety and depression demonstrated a similar degree of relationship (R = 0.29) with the severity of dysmenorrhea, followed by stress (R = 0.28). Self-esteem showed a weak negative relationship with the severity of dysmenorrhea (R = -0.07), indicating that the severity of dysmenorrhea decreases as the participants’ self-esteem scores increase.

**Fig 2:**
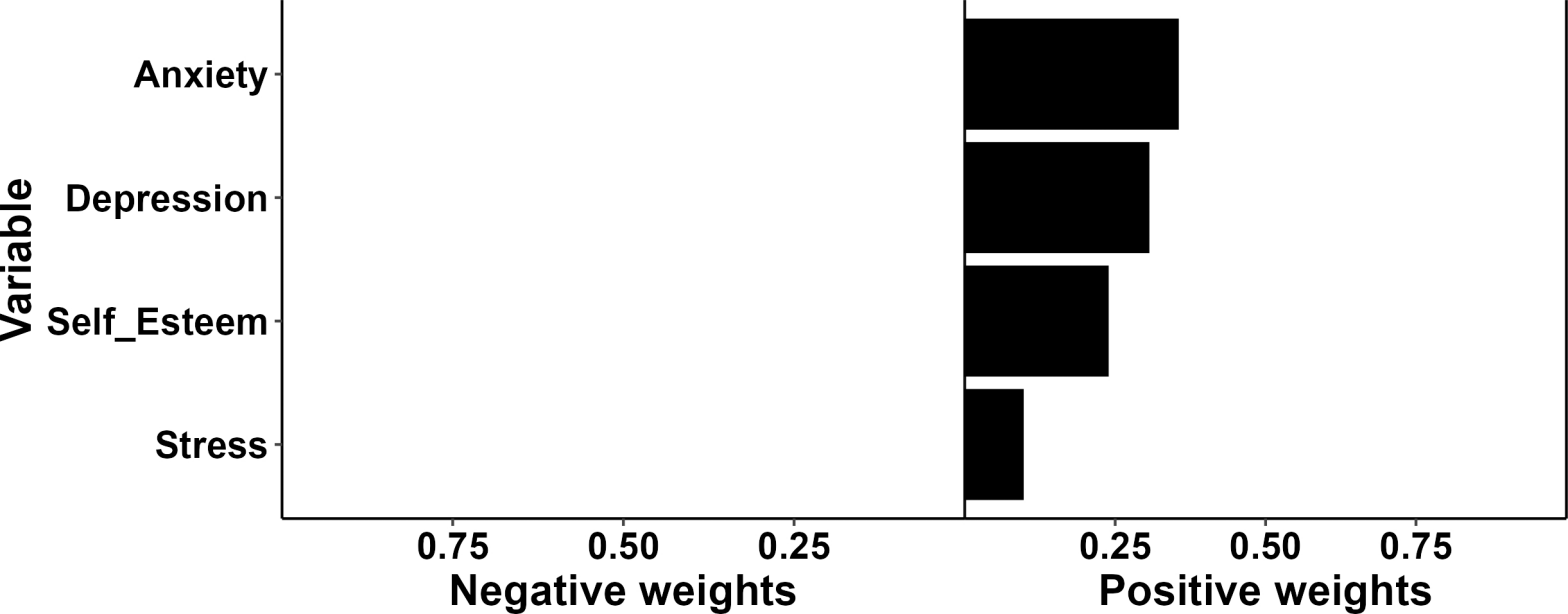
Plot of mental disorders weights using QGC.

The results of relative influence of each of the common mental disorders, as well as the combined influence of the four common mental disorders on the severity of dysmenorrhea are shown in Table 3. An average increase of 1.17 (95% CI: -0.28 – 2.06) in the severity of dysmenorrhea per quartile difference was associated with the combined influence of the four common mental disorders on the severity of dysmenorrhea. Anxiety showed the highest relative influence on the severity of dysmenorrhea, accounting for 36.0% of the influence (weight: 0.36), followed by depression (weight: 0.31), self-esteem (weight: 0.24), and stress (weight: 0.10) (Fig 2).

**Table 3:** Relative effect of common mental disorders on the severity dysmenorrhea using QGC and BKMR.

common mental disorders	Weights from QGC	PIPs from BKMR
Anxiety	0.36	0.62
Depression	0.31	0.50
Stress	0.10	0.29
Self-esteem	0.24	0.41
Collective association $\left(\beta \right)$	1.17 (95% CI: 0.28 – 2.06)	

Covariate: Age, students’ monthly allowance, menstrual cycle length, accommodation type, religion, and relationship with parent.

**Fig 3:**
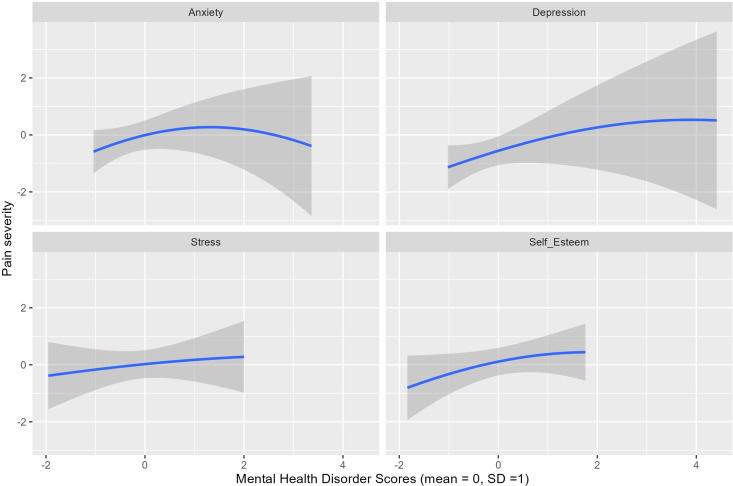
Mental health disorders effect pattern with 95% confidence interval.

Using BKMR, anxiety had the highest influence on the severity of dysmenorrhea, with the highest PIP (PIP: 0.62), followed by depression (PIP: 0.50), self-esteem (PIP: 0.41), and stress (PIP: 0.29). Fig 3 shows the patterns of each of the common mental disorders with the severity of dysmenorrhea. In general, the patterns aligned with the direction of influence indicated by the QGC results. There was an observed increasing non-monotonic influence of anxiety on the severity of dysmenorrhea, while depression, stress, and self-esteem had a linearly increasing influence on the severity of dysmenorrhea.

Stratified by the participants’ monthly allowance, relationship with parents, and type of accommodation, Table 4 shows the results of the subgroup-specific analysis. Using QGC, the collective influence of the four common mental disorders showed a lower influence on the severity of dysmenorrhea among participants with a sufficient monthly allowance (0.29, 95% CI: -1.034 – 1.61) compared to participants with an insufficient monthly allowance (2.02, 95% CI: 1.05 – 2.99). Anxiety showed the highest positive influence on the severity of dysmenorrhea with the highest weight (weight: 0.87) among participants with a sufficient monthly allowance, while depression (weight: 0.38) had the highest positive influence on the severity of dysmenorrhea, followed by self-esteem (weight: 0.33), among participants with an insufficient monthly allowance.

**Table 4:** Relative effect of common mental disorders to severity of dysmenorrhea from QGC and BKMR stratified by student allowance, relationship with parent and type of accommodation.

Common Mental Disorders	Student allowance^1^			
	Weights from QGC		PIPs from BKMR	
	Sufficient	Insufficient	Sufficient	Insufficient
Anxiety	0.87	0.17	0.40	0.94
Depression	0.13	0.38	0.35	0.48
Stress	−0.98	0.13	0.33	0.93
Self-esteem	−0.02	0.33	0.37	0.92
Collective effect $\left(\beta \right)$	0.29 (95% CI: -1.03 – 1.61)	2.02 (95% CI: 1.05 – 2.99)		
**Common Mental Disorders**	**Student relationship with parent** ^2^			
	**Weights from QGC**		**PIPs from BKMR**	
	**Satisfied**	**Unsatisfied**	**Satisfied**	**Unsatisfied**
Anxiety	0.26	−0.77	0.76	0.42
Depression	0.52	0.56	0.44	0.38
Stress	−1	−0.24	0.17	0.48
Self-esteem	0.22	0.44	0.30	0.67
Collective effect $\left(\beta \right)$	1.01 (95% CI: 0.31 – 1.72)	1.25 (95% CI: -0.03 – 2.53)		
**Common Mental Disorders**	**Student accommodation** ^3^			
	**Weights from QGC**		**PIPs from BKMR**	
	**In campus**	**Off-campus**	**In campus**	**Off-campus**
Anxiety	0.47	0.15	0.52	0.56
Depression	0.06	0.22	0.28	0.65
Stress	0.20	0.35	0.22	0.67
Self-esteem	0.27	0.28	0.28	0.50
Collective effect $\left(\beta \right)$	0.99 (95% CI: 0.06 – 1.92)	2.58 (95% CI: -1.31 – 6.48)		

^1^Covariate: Age, menstrual cycle length, accommodation type, religion, and relationship with parent.

^2^Covariate: Age, students’ monthly allowance, menstrual cycle length, accommodation type, and religion.

^3^Covariate: Age, students’ monthly allowance, menstrual cycle length, religion, and relationship with parent.

In the analysis stratified by participants’ relationship with their parents, the collective influence of the four common mental disorders resulted in a 1.01 (95% CI: 0.31 – 1.72) increase in the severity of dysmenorrhea per quartile difference for participants with a satisfied relationship with their parents and a 1.25 (95% CI: -0.03 – 2.53) increase in the severity of dysmenorrhea per quartile change for participants with an unsatisfied relationship with their parents, respectively. Anxiety (weight: -0.77) and stress (weight: -0.24) showed a decrease in the severity of dysmenorrhea among participants with an unsatisfied relationship. Among participants who reported a satisfied relationship with their parents, anxiety had the highest PIP on the severity of dysmenorrhea (PIP: 0.76), followed by depression (PIP: 0.44). In contrast, among participants with unsatisfied relationships with their parents, self-esteem (PIP: 0.67) had the highest PIP on the severity of dysmenorrhea, followed by stress (PIP: 0.48).

The collective influence of the four common mental disorders showed a 0.99 (95% CI: 0.06 – 1.92) increase in the severity of dysmenorrhea for participants staying on campus and a 2.58 (95% CI: -1.31 – 6.48) increase in the severity of dysmenorrhea for participants staying off-campus. Anxiety had the highest relative influence on the severity of dysmenorrhea (weight: 0.47) among participants staying on campus and a 15% increase in severity of dysmenorrhea (weight: 0.15) among participants staying off-campus. Using the BMKR, anxiety showed the highest PIP of 0.52 among participants staying on campus and a PIP of 0.56 among participants staying off-campus, while stress exhibited the highest PIP of 0.67 among participants staying off-campus and a PIP of 0.22 among participants staying on campus.

## 4 Discussion

This study showed a positive relationship between common mental disorders and the severity of dysmenorrhea, except for self-esteem that showed a negative relationship. Among the four common mental disorders, anxiety had the highest influence on the severity of dysmenorrhea, followed by depression, self-esteem, and stress. Collectively, the four common mental disorders had a positive influence on the severity of dysmenorrhea. This suggests a possible connection between common mental disorders and severity of dysmenorrhea, although the underlying mechanisms is difficult to ascertain. The association of anxiety and depression with the severity of dysmenorrhea can be attributed to the heightened emotional distress and dysregulation commonly observed in individuals with depression and anxiety. These conditions may intensify the body’s responses to pain stimuli, leading to increased pain sensitivity and amplified pain perception. Furthermore, the subjective experience of menstruation pain may be further intensified by cognitive distortions and maladaptive coping mechanisms often seen in individuals with anxiety and depression.

Similar to previous studies [19,37], anxiety and depression was the main common mental disorders associated with the severity of dysmenorrhea in our study. This finding is contrary to a previous study that reported depression as having a more pronounced effect on severity of dysmenorrhea than anxiety [38]. In this study, anxiety showed a greater weight (0.36) and PIP (0.62) compared to depression (weight: 0.31, PIP: 0.50). It is plausible that individuals with higher anxiety scores are more likely to report increased dysmenorrhea pain. The likelihood of reporting a severe dysmenorrhea pain was found to be higher among participants with high anxiety scores.

This study also showed that stress might also have an influence on the severity of dysmenorrhea, albeit to a lesser extent than anxiety and depression. Stress is widely recognized for its ability to impede the pulsatile release of follicle-stimulating hormone and luteinizing hormone, thereby disrupting follicular development [39]. Given that the luteinized follicle post-ovulation is responsible for progesterone synthesis, stress-induced impairment in follicular development may potentially disrupt progesterone synthesis and release. Progesterone is believed to play a pivotal role in dysmenorrhea, as menstrual pain occurs exclusively in ovulatory cycles, and progesterone has demonstrated influence over the synthesis and binding of prostaglandins PGF_2_α__ and PGE_2_ [40]. Prostaglandins play a crucial role in regulating uterine muscle and vascular tone [41,42]. An imbalance in prostaglandin levels has been associated with the occurrence of dysmenorrhea [43]. These findings showed that stress may exert both primary and secondary influences on the severity of dysmenorrhea. A similar study by Tahir et al. that examined the influence of macronutrient intake, stress, and prostaglandin levels (PGF_2_α__) on the incidence of dysmenorrhea in adolescent [44], and by Bavil et al., reported a positive and significant relationship between occupational stress and severity of dysmenorrhea [45].

Participants who received an insufficient monthly allowance from their parents or guardians reported a higher collective influence on the severity of dysmenorrhea compared to those who received an adequate monthly allowance. Due to the prevalence of financial stress among students with lower incomes, participants with low monthly allowances may experience more severe dysmenorrhea. As previously mentioned, stress intensifies the severity of dysmenorrhea. Students with greater financial means have access to more stress-relieving activities and coping strategies that might alleviate the effects of common mental disorders. Furthermore, students with lower incomes may experience poorer health outcomes due to nutritional inadequacies.

The higher collective influence of common mental disorders on the severity of dysmenorrhea among participants with unsatisfactory relationships with their parents, compared to those with satisfied relationships, can be attributed to various interconnected factors. The psychological well-being of a student is significantly impacted by the nature of parent-child connections. Students who have loving and caring relationships with their parents are more likely to have better mental health, which can alleviate the intensity of the pain associated with dysmenorrhea. However, students with unsatisfactory relationships may lack a strong support system, leading to feelings of isolation and intensifying their psychological distress and physical symptoms.

Anxiety and stress had a relatively decreased influence on the severity of dysmenorrhea among participants with unsatisfactory relationships with their parents. For students in this category, anxiety may act as a coping mechanism that unintentionally reduces the perception of pain associated with dysmenorrhea. This could be due to heightened levels of emotional distress, which may cause individuals to focus more on their anxiety than on the physical symptoms of dysmenorrhea.

Several factors may contribute to the higher influence of common mental disorders on the severity of dysmenorrhea among students residing off-campus compared to their on-campus counterparts. Off-campus students may have limited access to peer support networks, recreational centers, and mental health services available on campus. This lack of support can lead to increased stress and feelings of loneliness, which in turn can worsen mental health and aggravate symptoms of dysmenorrhea. It is also plausible that factors such as time constraints, transportation issues, adopting poor dietary habits, and having fewer opportunities for physical activity might additionally affect the mental health of participants, and influence their assessment of the severity of dysmenorrhea.

This study does have some potential limitations. This study design is cross-sectional making it difficult to establish causality between explanatory variables and dependent outcome. The determination of causal relationship between the four common mental disorders and severity of dysmenorrhea would be more plausible if longitudinal data were collected. The generalizability of the study findings should be interpreted with caution as the data were collected from young medical students who likely had some background knowledge of dysmenorrhea and common mental disorders. Further studies involving a more diverse group of young women from various socio-economic backgrounds are recommended to better understand these associations across the general population. The self-report questionnaire was used for data collection in this study, which could account for over reporting bias. To gain a more comprehensive understanding of the mental health status of the participants, future studies should consider mixed methods with opportunity to probe for specific response.

In conclusion, this study showed a positive collective influence of the four common mental disorders on the severity of dysmenorrhea among female medical students. It underscores the importance of prioritizing the mental health and well-being of female students by university governing councils and lecturers. This can be achieved through the implementation of mental health support programs and interventions aimed at addressing anxiety and depression, which have been identified as having a significant impact on the severity of dysmenorrhea. By addressing mental health concerns, universities can promote a supportive and conducive environment for female students to thrive academically and personally.

## Supporting information

S1 File Questionnaire for assessing the effect of mental health on the severity of dysmenorrhea in women. (DOCX)
