# Supplementary material for: Association between common mental disorders and the severity of dysmenorrhea among female medical students at the University of Ibadan, Nigeria
Source: PLOS Glob Public Health. 2025 Apr 23;5(4):e0004492. doi: 10.1371/journal.pgph.0004492 (PMC12017577; doi:10.1371/journal.pgph.0004492)
Supplement: S1 File — (DOCX) [file pgph.0004492.s001.docx]

**Questionnaire**

**QUESTIONNAIRE FOR ASSESSING THE EFFECT OF MENTAL HEALTH ON THE SEVERITY OF DYSMENORRHEA IN WOMEN**

Good day Ma,

We are a group of medical students currently collecting information on the mental health impact of dysmenorrhea in women. We would like to ask you some questions that would aid this research. We would collect information on; socio-demographic, pattern of menstruation, assessment of anxiety, assessment of depression, assessment of stress and assessment of self-esteem.

All information collected will be kept confidential. We will not ask for your name, address or any other information that can identify you. We hope you will be able to help us by participating in this research. Your participation in this research is voluntary and would be appreciated and respected.

Are you willing to participate? A. Yes B. No

If no, please state your reason ____________________________________________________

If yes, please answer each question as truthfully as possible and seek clarifications wherever needed. An interviewer is present to assist you and if you have any question, please do not hesitate to ask.

If you have any enquires about this study, you can contact the Corresponding Author via phone number 08064810347 or email [bvcadeyeye@gmail.com](mailto:bvcadeyeye@gmail.com)

Thank you.

**SECTION 1: SOCIO-DEMOGRAPHIC INFORMATION**

|  | **Question and Filter** | **Coding Category** | **Response Column** |
| --- | --- | --- | --- |
| Q1.1 | Age *(as at last birthday in years)* | \|_____\|_____\| | |
| Q1.2 | Academic level? | 100L = 01  200L = 02  300L = 03  400L = 04  500L = 05  600L = 06  600BL = 07 | \|_____\|_____\| |
| Q1.3 | Program you are currently studying? | Medicine and Surgery = 01  Dentistry = 02 | \|_____\|_____\| |
| Q1.4 | Where do you live? | In campus = 01  Alone off campus *(rented apartment)* = 02  Off campus with family = 03 | \|_____\|_____\| |
| Q1.5 | Religion | Christianity = 01  Islam = 02  Traditional = 03  Others = 04 | \|_____\|_____\| |
| Q1.6 | Tribe | Yoruba = 01  Hausa = 02  Igbo = 03  Others = 04 | \|_____\|_____\| |
| Q1.7 | Marital Status | Single = 01  Married = 02  Separated = 03  Divorced = 04  Widowed = 05  Cohabiting *(with your sexual partner)* = 06 | \|_____\|_____\| |
| Q1.8 | Amount of physical activity weekly | Only baseline activity = 01  Less than 2½ hours of exercise weekly = 02  2½ - 5 hours of exercise weekly = 03  More than 5 hours of exercise weekly = 04 | \|_____\|_____\| |
| Q1.9 | Father’s highest level of education | Post-graduate = 01  Tertiary = 02  Secondary = 03  Primary = 04  Islamic = 05  None = 06 | \|_____\|_____\| |
| Q1.10 | Mother’s highest level of education | Post-graduate = 01  Tertiary = 02  Secondary = 03  Primary = 04  Islamic = 05  None = 06 | \|_____\|_____\| |
| Q1.11 | Family type | Nuclear = 01  Extended = 02 | \|_____\|_____\| |
| Q1.12 | Family structure | Monogamous = 01  Polygamous = 02 | \|_____\|_____\| |
| Q1.13 | How often do you speak with your parents on telephone? | Everyday = 01  Sometimes a week = 02  Once a week = 03  Sometimes a month = 04  Sometimes a year = 05  Never = 06 | \|_____\|_____\| |
| Q1.14 | How satisfied are you with your relationship with your parents? | Very satisfied = 01  Somewhat Satisfied = 02  Indifferent = 03  Unsatisfied = 04  Extremely Unsatisfied = 05 | \|_____\|_____\| |
| Q1.15 | Did your parents ever split up or divorce? | Yes = 01  No = 02 | \|_____\|_____\| |
| Q1.16 | Do your mother and father live together? | Yes = 01  No = 02 | \|_____\|_____\| |
| Q1.17 | If parents do not live together, who do you stay with presently? | Father = 01  Mother = 02 | \|_____\|_____\| |
| Q1.18 | Source of income to school | Father = 01  Mother = 02  Both parent = 03  Other relatives = 04  Self = 05  Others = 06 | \|_____\|_____\| |
| Q1.19 | What is your average allowance per month? | \|_______________________________\| | |
| Q1.20 | Do you have any female sibling? | Yes = 01  No = 02 | \|_____\|_____\| |
| Q1.21 | Are you currently breastfeeding? | Yes = 01  No = 02 | \|_____\|_____\| |

**SECTION 2: PATTERN OF MENSTRUATION**

|  | **Question and Filter** | **Coding Category** | **Response Column** |
| --- | --- | --- | --- |
| Q2.1 | Are you having cyclical menstruation? | Yes = 01  No = 02 | \|_____\|_____\| |
| Q2.2 | How old were you *(in years)* when you first had your menstruation *(monthly vaginal bleeding)?* | \|_____\|_____\| | |
| Q2.3 | Who did you inform when you had your first menstruation? | Mother = 01  Father = 02  Parents (mother and father at the same time) = 03  Siblings = 04  Siblings = 05  Friend = 06  Others = 07 | \|_____\|_____\| |
| Q2.4 | What is your average menstrual cycle length *(duration from the first day of your last menses to the time you have your next one) (please indicate in days)* | \|_____\|_____\| | |
| Q2.5 | How long do you normally menstruate? *(Please indicate in days)* | \|_____\|_____\| | |
| Q2.6 | Do you use sanitary pads or tampons during menstruation? | Yes = 01  No = 02 | \|_____\|_____\| |
| If “No” to Q2.6, skip to Q2.10 | | | |
| Q2.7 | On the average, how many sanitary pads or tampons do you use during your menstrual period? | \|_____\|_____\| | |
| Q2.8 | Has there been any other time when you used more than the number mentioned above? | Yes = 01  No = 02 | \|_____\|_____\| |
| Q2.9 | If yes, how many more? | \|_____\|_____\| | |

Tick appropriately.

Q2.10 How many times did you need to get out of bed in the middle of night *(or during sleep hours)* to change your sanitary pads/tampons?

|  |  | Never | 1-3 times | 4-6 times | 7-10 times | 11 times or greater |
| --- | --- | --- | --- | --- | --- | --- |
| a. | Past 3 months |  |  |  |  |  |
| b. | Past 6 months |  |  |  |  |  |
| c. | Past 12 months |  |  |  |  |  |

Q2.11 During the past 3 months, how would you describe your periods?

(01) Very Light (02) Light (03) Moderate (04) Heavy (05) Very Heavy

Q2.12 How many times have you had an episode of bleeding that soaked through your “outer” clothes *(pants, skirt, dress)*?

|  |  | Never | 1-3 times | 4-6 times | 7-10 times | 11 times or greater |
| --- | --- | --- | --- | --- | --- | --- |
| a. | Past 3 months |  |  |  |  |  |
| b. | Past 6 months |  |  |  |  |  |
| c. | Past 12 months |  |  |  |  |  |

Q2.13 How many times did you pass blood clots *(clumps of blood)?*

|  |  | Never | 1-3 times | 4-6 times | 7-10 times | 11 times or greater |
| --- | --- | --- | --- | --- | --- | --- |
| a. | Past 3 months |  |  |  |  |  |
| b. | Past 6 months |  |  |  |  |  |
| c. | Past 12 months |  |  |  |  |  |

|  | **Question and Filter** | **Coding Category** | **Response Column** |
| --- | --- | --- | --- |
| Q2.14 | Have you ever experienced lower abdominal pain during menstruation? | Yes = 01  No = 02 | \|_____\|_____\| |
| If No to question Q2.14, go to question Q2.27 | | | |
| Q2.15 | How old were you when you had the first painful menstruation? | \|_____\|_____\| | |
| Q2.16 | When was the last time you had lower abdominal pain during menstruation? | During the last menstrual period = 01  Within the last 6 months before last menstrual period = 02  Between 6 months to 1 year before last menstrual period = 03  Greater than 1 year before last menstrual period = 04 | \|_____\|_____\| |
| Q2.17 | How frequent is your menstrual pain? | Every month = 01  Once in 3 months = 02  Once in 6 months = 03  Once yearly = 04  Less than once yearly = 05 | \|_____\|_____\| |
| Q2.18 | On a scale of 0 to 10 where **0** means no pain and **10** means the most severe pain ever imagined, rate your pain | \|_____\|_____\| | |
| Q2.19 | List all the options that describe your pain | My pain does not prevent me from doing my daily activity = 01  My pain is so severe that I cannot do my daily routine without using medication = 02  My pain is so severe that I usually get admitted into the hospital = 03 | \|_____\|_____\|  \|_____\|_____\|  \|_____\|_____\| |
| Q2.20 | Do you use anything for the pain? | Yes = 01  No = 02 | \|_____\|_____\| |
| If "yes" to Q2.20 | | | |
| Q2.21 | What do you use for the pain? | Mild analgesics *(paracetamol)* = 01  Moderate analgesics *(codeine, Non-steroidal anti-inflammatory drugs)* = 02  Strong analgesics *(morphine)* = 03 | \|_____\|_____\| |
| Q2.22 | How is the pain related to your menstruation? | It occurs before the menstruation starts = 01  It gets worse as menstruation begins = 02  It is the same throughout menstruation = 03 | \|_____\|_____\| |
| Q2.23 | Is there any other member of your family that experiences cyclical pain? | Yes = 01  No = 02 | \|_____\|_____\| |
| If "no" to Q2.23 skip Q2.24 | | | |
| Q2.24 | Kindly indicate which member(s) of your family by selecting one or more options | Mother = 01  Sister = 02  Aunty = 03 | \|_____\|_____\| |
| Q2.25 | Are there non-pharmacological techniques you use to reduce the severity of the pain? | Yes = 01  No = 02 | \|_____\|_____\| |
| If "yes" to Q2.25 | | | |
| Q2.26 | What are those techniques? | Swimming = 01  Talking lime = 02  Taking hot water = 03  Other = 04 | \|_____\|_____\| |
| Q2.27 | How often do you miss school because of your periods? | Never = 01  Rarely = 02  Sometimes = 03  Always = 04 | \|_____\|_____\| |
| Q2.28 | How many days in a cycle do you have to miss school because of your period? | \|_____\|_____\| | |
| Q2.29 | What about your period causes you to miss school? | Pain = 01  Heavy menstruation = 02  Nausea and vomiting = 03  Others = 04 | \|_____\|_____\| |

**SECTION 3 – ASSESSMENT OF ANXIETY**

Below is a list of common symptoms of anxiety. Please carefully read each item in the list. Indicate how much you have been bothered by that symptom, including today, by ticking the corresponding space in the column next to each symptom.

|  | **Questions** | **Not at all** | **Several days** | **More than half the days** | **Nearly every day** |
| --- | --- | --- | --- | --- | --- |
| Q3.1 | Over the last 6 menstrual cycles how often have you been bothered by the following problems? |  |  |  |  |
| Q3.2 | Feeling nervous, anxious, or on edge |  |  |  |  |
| Q3.3 | Not being able to stop or control worrying |  |  |  |  |
| Q3.4 | Worrying too much about different things |  |  |  |  |
| Q3.5 | Trouble relaxing |  |  |  |  |
| Q3.6 | Being so restless that it is hard to sit still |  |  |  |  |
| Q3.7 | Becoming easily annoyed or irritable |  |  |  |  |
| Q3.8 | Feeling afraid, as if something awful might happen |  |  |  |  |

**SECTION 4 –ASSESSMENT OF DEPRESSION**

Kindly tick the option that best applies to you over the past 6 menstrual cycles.

|  | **Question and filter** | **Response Column** |
| --- | --- | --- |
| Q4.1 | I do not feel sad = 01  I feel sad = 02  I am sad all the time and I can't snap out of it = 03  I am so sad and unhappy that I can't stand it = 04 | \|_____\|_____\| |
| Q4.2 | I am not particularly discouraged about the future = 01  I feel discouraged about the future = 02  I feel I have nothing to look forward to = 03  I feel the future is hopeless and that things cannot improve = 04 | \|_____\|_____\| |
| Q4.3 | I do not feel like a failure = 01  I feel I have failed more than the average person = 02  As I look back on my life, all I can see is a lot of failures = 03  I feel I am a complete failure as a person = 04 | \|_____\|_____\| |
| Q4.4 | I get as much satisfaction out of things as I used to = 01  I don't enjoy things the way I used to = 02  I don't get real satisfaction out of anything anymore = 03  I am dissatisfied or bored with everything = 04 | \|_____\|_____\| |
| Q4.5 | I don't feel particularly guilty = 01  I feel guilty a good part of the time = 02  I feel quite guilty most of the time = 03  I feel guilty all of the time = 04 | \|_____\|_____\| |
| Q4.6 | I don't feel I am being punished = 01  I feel I may be punished = 02  I expect to be punished = 03  I feel I am being punished = 04 | \|_____\|_____\| |
| Q4.7 | I don't feel disappointed in myself = 01  I am disappointed in myself = 02  I am disgusted with myself = 03  I hate myself = 04 | \|_____\|_____\| |
| Q4.8 | I don't feel I am any worse than anybody else = 01  I am critical of myself for my weaknesses or mistakes = 02  I blame myself all the time for my faults = 03  I blame myself for everything bad that happens = 04 | \|_____\|_____\| |
| Q4.9 | I don't have any thoughts of killing myself = 01  I have thoughts of killing myself, but I would not carry them out = 02  I would like to kill myself = 03  I would kill myself if I had the chance = 04 | \|_____\|_____\| |
| Q4.10 | I don't cry any more than usual = 01  I cry more now than I used to = 02  I cry all the time now = 03  I used to be able to cry, but now I can't cry even though I want to = 04 | \|_____\|_____\| |
| Q4.11 | I am no more irritated by things than I ever was = 01  I am slightly more irritated now than usual = 02  I am quite annoyed or irritated a good deal of the time = 03  I feel irritated all the time = 04 | \|_____\|_____\| |
| Q4.12 | I have not lost interest in other people = 01  I am less interested in other people than I used to be = 02  I have lost most of my interest in other people =03  I have lost all of my interest in other people = 04 | \|_____\|_____\| |
| Q4.13 | I make decisions about as well as I ever could = 01  I put off making decisions more than I used to = 02  I have greater difficulty in making decisions more than I used to = 03  I can't make decisions at all anymore = 04 | \|_____\|_____\| |
| Q4.14 | I don't feel that I look any worse than I used to = 01  I am worried that I am looking old or unattractive = 02  I feel there are permanent changes in my appearance that make me look unattractive = 03  I believe that I look ugly = 04 | \|_____\|_____\| |
| Q4.15 | I can work about as well as before = 01  It takes an extra effort to get started at doing something = 02  I have to push myself very hard to do anything = 03  I can't do any work at all = 04 | \|_____\|_____\| |
| Q4.16 | I can sleep as well as usual = 01  I don't sleep as well as I used to = 02  I wake up 1-2 hours earlier than usual and find it hard to get back to sleep = 03  I wake up several hours earlier than I used to and cannot get back to sleep = 04 | \|_____\|_____\| |
| Q4.17 | I don't get more tired than usual = 01  I get tired more easily than I used to = 02  I get tired from doing almost anything = 03  I am too tired to do anything = 04 | \|_____\|_____\| |
| Q4.18 | My appetite is no worse than usual = 01  My appetite is not as good as it used to be = 02  My appetite is much worse now = 03  I have no appetite at all anymore = 04 | \|_____\|_____\| |
| Q4.19 | I haven't lost much weight, if any, lately = 01  I have lost more than 2.5kg without intention = 02  I have lost more than 5kgs without intention = 03  I have lost more than 7kgs without intention = 04 | \|_____\|_____\| |
| Q4.20 | I am no more worried about my health than usual = 01  I am worried about physical problems like aches, pains, upset stomach, or constipation = 02  I am very worried about physical problems and it's hard to think of much else = 03  I am so worried about my physical problems that I cannot think of anything else = 04 | \|_____\|_____\| |
| Q4.21 | I have not noticed any recent change in my interest in sex = 01  I am less interested in sex than I used to be = 02  I have almost no interest in sex = 03  I have lost interest in sex completely = 04 | \|_____\|_____\| |

**SECTION 5 – ASSESSMENT OF STRESS LEVEL**

For each question choose the option that best applied to you

|  | **Question** | **Never** | **Almost never** | **Fairly often** | **Very Often** |
| --- | --- | --- | --- | --- | --- |
| Q5.1 | In the last month, how often have you been upset because of something that happened unexpectedly? |  |  |  |  |
| Q5.2 | In the last month, how often have you felt that you were unable to control the important things in your life? |  |  |  |  |
| Q5.3 | In the last month, how often have you felt nervous and stressed? |  |  |  |  |
| Q5.4 | In the last month, how often have you felt confident about your ability to handle your personal problems? |  |  |  |  |
| Q5.5 | In the last month, how often have you felt that things were going your way? |  |  |  |  |
| Q5.6 | In the last month, how often have you found that you could not cope with all the things that you had to do? |  |  |  |  |
| Q5.7 | In the last month, how often have you been able to control irritations in your life? |  |  |  |  |
| Q5.8 | In the last month, how often have you felt that you were on top of things? |  |  |  |  |
| Q5.9 | In the last month, how often have you been angered because of things that happened that were outside of your control? |  |  |  |  |
| Q5.10 | In the last month, how often have you felt difficulties were piling up so high that you could not overcome them? |  |  |  |  |

**SECTION 6 – ASSESSMENT OF SELF ESTEEM**

Below is a list of statements dealing with your general feelings about yourself over the last 6 menstrual cycles. Please indicate how strongly you agree or disagree with each statement.

|  | **Question** | **Strongly Agree** | **Agree** | **Disagree** | **Strongly Disagree** |
| --- | --- | --- | --- | --- | --- |
| Q6.1 | On the whole, I am satisfied with myself |  |  |  |  |
| Q6.2 | At times I think I am no good at all |  |  |  |  |
| Q6.3 | I feel that I have a number of good qualities |  |  |  |  |
| Q6.4 | I am able to do things as well as most other people |  |  |  |  |
| Q6.5 | I feel I do not have much to be proud of |  |  |  |  |
| Q6.6 | I certainly feel useless at times |  |  |  |  |
| Q6.7 | I feel that I'm a person of worth, at least on an equal plane with others |  |  |  |  |
| Q6.8 | I wish I could have more respect for myself |  |  |  |  |
| Q6.9 | All in all, I am inclined to feel that I am a failure |  |  |  |  |
| Q6.10 | I take a positive attitude toward myself |  |  |  |  |
